# Pathopolitics: Pathologies and Biopolitics of PrEP

**DOI:** 10.3389/fsoc.2020.00053

**Published:** 2020-08-12

**Authors:** Tankut Atuk

**Affiliations:** Department of Gender, Women and Sexuality Studies, University of Minnesota Twin Cities, St. Paul, MN, United States

**Keywords:** PrEP, HIV, pathologies of power, Corporate Social Responsibility (CSR), Gilead Sciences, Inc., biopolitics

## Abstract

This paper unveils the pathologies that are produced and sustained by the pharmaceutical industry, specifically by Gilead Sciences, Inc. Broadly defined, pathopolitics is the politics of treating and/or reproducing pathologies. This paper examines pathopolitics in the context of PrEP, or *pre-exposure prophylaxis*, an antiretroviral medicine that prevents HIV transmission. Although Gilead promises to prevent a pathology through PrEP, it reproduces social and biological pathologies by exposing certain people to higher risks of infections and diseases, thus epitomizing the operating logic of the pharmaceutical industry: that life is protected only insofar as it offers surplus economic and social value. This essay raises three fundamental sets of questions: (1) What are the techniques and mechanics of pathopolitics? (2) How does the pharmaceutical industry produce and exploit surplus value? (3) What is the nature of the relationship between the pharmaceutical citizenship and pathopolitics?

## Introduction

“Contemporary biopolitics is *risk politics*.”

—Nicholas Rose (2001, 1: emphasis original)

Following the beginning of the epidemic in the 1980s, HIV and AIDS have become a central field of biopolitical interventions and biomedical surveillance. Informed by a pseudo-scientific homophobia, the biopolitics of HIV has targeted less the ending of the epidemic than disciplining gay men and curing them of alleged pathological practices. Halperin (2015, p. 206) notes that by the end of the 1980s, epidemiologists considered changes in the sexual behaviors of gay men as the “most profound modifications of personal health-related behaviors ever recorded.” The fear of AIDS of course played a crucial role in this. The Western public health rhetoric, likewise, has often disciplined deviant sexualities by spreading the fear of HIV transmission (Tewksbury, 2003; Holmes and O'Byrne, 2010). The same fear is adamantly kept alive today to surveil and extract profit from gay men's bodies, HIV+ or not (Race, 2009). In 21st century, “biopolitics becomes bioeconomics driven by the search for what Catherine Waldby (2002) has termed ‘biovalue’: the production of a surplus out of vitality itself” (Rose, 2001, p. 15).

The convergence of biopolitics into bioeconomics and the subsequent extraction of value out of “vitality itself” was only possible as a result of a radical transformation of the meanings of health, disease, and risk. With developing medical technologies, “our increased knowledge about nutrition, disease, and medicine,” Dean wrote, “has not produced a greater sense of security but, on the contrary, a heightened sense of risk” (2009, p. 62). Dumit (2012, p. 1) too noted.

“Health in America today is defined by a double insecurity: never being sure enough about the future—always being at risk—and never knowing enough about what you could and should be doing. Paradoxically, the insecurity continues to grow despite there being an equal growth in the amount of medicine consumed each year—as if the more we know, the more we fear; and the more we fear, the more preventive actions and medications we need to take.”

Dumit (2012) also called our attention to how the pharmaceutical industry redefined (surplus) health to create new markets and generate demand for new medicine. Health today means not preventing diseases but reducing risk since everyone is assumed to be “inherently ill.” Being inherently ill hits gay men close to home, for they have long been conceived and have conceived themselves as always already sick, even before the epidemic.

Since 2012, the pervasive commodification and regulation of queer sexualities and bodies has taken a new form with the expansion of the use of antiretroviral medicine for HIV negative people. The new definition of health as risk prevention requires that one is “PrEPared” all the time, as many gay men like to put it on the social media. This, Thomann (2018) argues, indexes the pharmaceuticalisation of the neoliberal sexual actor, as self-responsible as self-interested and rational, who is encouraged to respond to HIV risk pre-emptively through PrEP, trade name Truvada, an antiretroviral (ARV) medicine manufactured by the transnational pharmaceutical company Gilead since 2004. Since 2012, it has been used as pre-exposure prophylaxis, the scientific term from which the more common, more euphonic, and market-friendly abbreviation “PrEP” is derived. Truvada alone, when taken daily or as otherwise recommended, is more than 99% effective in providing protection against HIV (Grant et al., 2010; Anderson et al., 2012).

In this essay, I focus on the biopolitics and pathologies of PrEP. Foucault developed his ideas on biopolitics that first appeared on the first volume of *The History of Sexuality* during a series of lectures gathered under the name of *Society Must Be Defended*. There he explained, “Biopolitics deals with the population, with the population as a political problem, as a problem that is at once scientific and political, as a biological problem and as power's problem” (2003, p. 245). By biopolitics, I specifically refer to (a) governance of bodies in the name of health and (b) management of life chances, that is, manipulating who will be protected from and exposed to risk. Medical anthropologist and physician Paul Farmer chooses the phrase *pathologies of power* to describe this latter function of biopolitics invested in determining “who will suffer abuse and who will be shielded from harm” (2003, p. 7).

I offer the term *pathopolitics*^1^ to draw attention to the pathological nature of biopolitics under the pharmaceutical industry. The leading actor of pathopolitics is the pharmaceutical industry, commonly known as the Big Pharma, whose *raison d'etre* is curing pathologies, even though it survives through the reproduction of both biological and social pathologies. Pathopolitics is essentially biopolitics enacted by the pharmaceutical industry; in other words, it is a particular way of dealing with the population as a political and medical problem that needs to be distinguished from biopolitics writ large. Instead of relying on governmental or non-governmental techniques of managing life and death, pathopolitics operates primarily through corporate strategies of risk distribution.

Pathos in ancient Greek means, among other things, suffering. Therefore, pathology (*pathos-logia)* by definition signals suffering and pathopolitics can be defined in terms of ending and/or perpetuating pathologies as well as the suffering they cause. Paradoxically, the contemporary pharmaceutical industry prevents some pathologies while reproducing others—indeed, sometimes it produces certain pathologies precisely to treat others. Like biopolitics, pathopolitics makes live, lets die, and makes die, but in a slightly different fashion. Administering enough or too much medicine into bodies or depriving bodies of the necessary medicine is how pathopolitics determines who will be exposed to and protected from risk. While biopolitics can produce death in numerous distinct ways, death under pathopolitics will only take the shape of a disease or a pathology, which can mostly be prevented.

There are essentially two problems with the pharmaceutical industry and its pathopolitics: on the one hand, it penetrates too deeply into people's lives and bodies and turns them into a not-so-fictitious capital. The human body and its biological functions are made into physical assets that keep producing profit as long as they are alive (and, in our case, aroused). In this instance, the omnipresence and omnipotence of the pharmaceutical regime is what renders it extremely violent. On the other hand, the problem is its absence: the pharmaceutical regime is not equally concerned about populations whose medicalization does not promise an inexhaustible source of profit. The violence occurs in this case not from being subjected by/to pharmaceutical regimes but from being ignored/erased by them. Pharmaceutical (mis)management of bodies is a double-edged sword invested in the “overtreatment of some and undertreatment of others” (Tomes, 2016, p. 2). The phenomenon is also carefully documented in *Global Pharmaceuticals* by Petryna et al. (2006) who described the constitutive contradiction of pharmaceutical markets in terms of *access* vs. *excess*.

Instead of looking at how PrEP intervenes in the prevention of pathologies as has been done abundantly by public health and HIV experts, I examine pathologies that are produced and sustained by the pharmaceutical industry in tandem with widespread structural inequalities. To accomplish this, I raise three interrelated questions: (1) What are the techniques and mechanics of pathopolitics? (2) How does the pharmaceutical industry produce and exploit surplus value? (3) What is the nature of the relationship between the pharmaceutical citizenship and pathopolitics? In response to these questions, I argue that although Gilead Sciences, Inc. promises to prevent a pathology through PrEP, it reproduces social and biological pathologies that expose certain people to higher risks of infections and diseases. This happens in three fundamental ways: by setting exorbitant drug prices, halting generics, and relocating pathologies to developing countries. Here, I also claim that PrEP lays bare the constitutive failure, or the operating logic, of the pharmaceutical industry: life is only worth protecting from risk as long as it can offer surplus value. Finally, I make the case that uncritical advocacy and consumption of a drug in the name of health or pleasure can inadvertently reproduce pathopolitics, for it will invisibilize the unjust—or, to put it differently, pathological—mechanics through which risk is distributed.

In this article, I interpret the violence enacted by pathopolitics on those undertreated as an instance of slow violence, so normalized, pervasive and pernicious that it rarely makes into news. Nixon (2011) employs the term slow violence to account for the environmental damage both on nature and human life, which is readily ignored because it is neither spectacular nor instantaneous. As a result of slow violence, people are perpetually debilitated—living each day without necessary medications or care brings them one step closer to illness. Slow violence brings slow death, which Berlant notes, “shapes our particular biopolitical phase: mainly, people do live in it, just not very well” (2007, p. 780). To paraphrase Foucault, the question is if the pharmaceutical industry's objective is essentially to make live, how can it let die? (2003, p. 254).

## Preventing HIV at the Cost of $24,000

“With its vested interest in biological catastrophism, neoliberalism is similarly intent on profiting from the ‘unregulated’ distribution of life chances, however extreme.”

Cooper (2011, p. 11)

When it comes to the production and sale of ARVs, Gilead is the largest and richest company and Truvada is one of its most important sources of profit, bringing in more than US $ 3 billion each year (Langreth and Brown, 2019). The development of new and better ARV medicines led Gilead to create alternative markets for its old compounds to extend patent protection (Spieldenner, 2016), which is commonly known as “evergreening.” Truvada has been a part of anti-retroviral treatment of HIV since 2004. Later, in 2012, it was approved by the Food and Drug Administration (FDA) as PrEP. It consists of Emtricitabine and Tenofovir, which together inhibit the replication of HIV and thereby controls its growth. The first successful PrEP trials (iPrEx) were initiated in Peru and Ecuador in 2007, and were extended to Brazil, South Africa, Thailand, and the U.S. In 2010, the first set of results demonstrated that Truvada provides protection from HIV infection by up to 99% when taken daily (Grant et al., 2010; Anderson et al., 2012). For public health authorities this was a harbinger of a new era in HIV prevention and for many in the queer community it heralded a sexual revolution, which offered the chance to say goodbye to condoms, which is not necessarily antithetical to the principles of public health and HIV prevention (Brisson et al., 2019; Rojas Castro et al., 2019). Three decades after the AIDS epidemic, gay and trans people were once again able to enjoy sex without latex barriers and with virtually no risk of HIV transmission. This found widespread criticism from influential figures of the early AIDS movement such as Larry Kramer and Michael Weinstein, the president of the AIDS Healthcare Foundation. While the former considered PrEP as an erasure of the history of AIDS and the end of the fight against HIV (Healy, 2014)^2^, the latter suspected an ominous increase in the transmission of sexually transmitted infections (STIs) (Ryan, 2017).

Gilead, who spent more than 100 million dollars in 2017 alone on advertising Harvoni, a Hepatitis C medicine, spent merely several hundred thousand dollars per year on promoting PrEP (Fitzsimons, 2018). This considerably small-scale marketing strategy of Gilead can be interpreted on two registers: first, as I will mention in more detail below, Gilead sought to portray PrEP as a public health intervention and not a commercial tool. Second, gay men, public health experts, and government agencies took it on themselves to popularize PrEP. In 2014, the CDC suggested half a million of uninfected Americans should go on PrEP as an HIV prevention strategy. In 2019, Gilead, the producer of Truvada, announced in its publicly accessible second quarterly earning results that more than 213,000 Americans are on PrEP and the numbers are rapidly growing. Nevertheless, PrEP uptake has counterintuitively been slow in spite of its often-cited (by Gilead and CDC) public health potentials and it remains inaccessible to those who need it most (CDC., 2019). Truvada for PrEP in the US costs approximately $24,000 per year plus the expenses of visits and obligatory tests every 3 months. The exorbitant prices are commonly justified by citing the expenses of research and development even though “after tax deductions only about 1.3 percent of the money that the industry spends actually goes into basic research, the type of research that leads to new medications” (Lexchin, 2018, p. 2). Moreover, the research necessary for the discovery of new drugs is usually undertaken by universities or governments and funded by philanthropic organizations or the NIH. The Democratic congresswoman Alexandria Ocacio-Cortez brought to public attention in May 2019 that the research that enabled the use of Truvada as PrEP was publicly funded through taxes^3^. Following is an excerpt from the testimony of Dr. Robert Grant, who is the leading scientist of the first successful PrEP trial:

I believe that the root cause of low PrEP access is the high price of the medication. PrEP can be manufactured and distributed, including a profit, for about $6 per person per month. Gilead charges more than $2,100 per person per month, a 35,000% markup. Gilead's prices continue to increase: Gilead has increased the price of Truvada 76% since I published evidence of PrEP efficacy in 2010, using US government funding. You might hear that “no one pays” the list price after discounts. This is not true […] In my experience, public health officials are reluctant to promote PrEP in their jurisdictions because of the high price of PrEP medications (House Committee on Oversight and Reform, 2019).

McKenney et al. (2017) demonstrated PrEP drug costs must be reduced to be a cost-effective and efficient prevention method. Along the same lines, Patel et al. (2017) noted insured people are four times as likely to use PrEP compared to the uninsured. Doblecki-Lewis et al. (2017) too pointed out that white people and people with health insurance are more likely to use PrEP. The biggest obstacle in providing PrEP for all is the absence of generics in the US (although they can be ordered from abroad). Gilead substantiates the popular belief that Big Pharma, infamous for morally questionable marketing techniques like patent interference and evergreening, has blood on its hands when it comes to generics. In 2018, the FDA published a list of pharmaceutical companies blocking the production of generics. Gilead secured a place on the list for preventing generics of Truvada among a few other medicines (FDA, 2018). Moreover, the company is accused of reaching agreements with potential generic manufacturers behind closed doors to halt generics (Rowl, 2019). When the unethical and rapacious actions of Gilead hit the fan, the company eventually announced the introduction of generic PrEP in the US in 2020. Nevertheless, the patient groups and activists are not thrilled about the news since Gilead will share the patent with a single manufacturer, Israel-based Teva, one of the pharmaceutical companies accused of fueling the opioid crisis in the U.S. (Lovelace, 2019). This is naturally not expected to result in a significant decrease in the price of Truvada due to the continuing monopoly over the patent. The timing of this announcement is highly suspect too: first, the patent of Truvada is already going to expire in 2021. Second, at the time of this writing, Gilead obtained approval for another medicine, Descovy, as PrEP (Fitzsimons, 2019). The company has been sued in the past few years for intentionally deferring the use of Descovy until Truvada's patent expires, even though the former is proven to be less toxic. This crystallizes the fundamental mechanics of pathopolitics: not only does Gilead perpetuate pathologies and suffering by making life-saving drugs inaccessible as a result of high prices and lack of generics, but also it openly causes those who take its drugs to suffer easily preventable life-threatening side-effects. This is a crucial point for one of the central claims this paper makes: in the next section, I will discuss how human life is protected only insomuch as it promises financial returns. Nonetheless, the intentional delaying of Descovy makes clear that even those whose lives can be capitalized are expandable within pathopolitics.

According to the data provided by the U.S. Department of Health & Human Services, populations disproportionately affected by HIV are gay men (and especially gay men of color), people of color, queer and trans people (of color), and IV substance users. Notwithstanding, studies showed those that are disproportionately affected by HIV also have greater difficulties accessing PrEP (and, treatment too): IV drug users (Guise et al., 2017), young transgender women (Wood et al., 2017), black men who have sex with men (MSM), transgender women (Hoots et al., 2016; Garnett et al., 2018), and male sex workers (Underhill et al., 2014) reported higher barriers to access PrEP. These studies reported that disparities in PrEP uptake stem from mistrust in the medical system, lack of information, limited awareness, lack of universal health care and high prices of pharmaceuticals. In 2017, after receiving widespread criticism by activist groups like ACT UP, Gilead broke the silence and finally admitted the racial disparities in the use of PrEP. The numbers shared by Gilead disclosed that the white population makes up 27% of new HIV incidents but 75% of PrEP users; whereas African-Americans and Hispanics respectively make up 44% and 23% of new cases but only 10 and 12% of PrEP uptake (Levin, 2017). Another set of results was released in March 2018:

In 2015, there were approximately 1.1 million Americans who could potentially benefit from PrEP: 500,000 African Americans, 300,000 Latinos, and 300,000 whites. However, analysis of available data on PrEP prescriptions finds that 7,000 prescriptions were filled at retail pharmacies or mail order services for African-Americans [that is, only 1%] and only 7,600 for Latinos [3%] during a similar time period (September 2015–August 2016) (CDC., 2018).

On the other hand, today women represent only 11.4% of current PrEP consumers (no racial or ethnic data is provided) (Levin, 2017). Although Gilead claims a growing increase in PrEP uptake, a set of recent studies still point out significant racial and gendered disparities (Golub, 2018; Kuehn, 2018; Caponi et al., 2019; Jenness et al., 2019).

These numbers would be confusing for someone who has recently watched Gilead's TV ads or visited Gilead's social media campaign *HealthySexuals*. Both are saturated with the images of queer people of color (POC), operating within a framework of public health and centering them as the targets of HIV **prevention**^4^. In a statement on its TV ads Gilead declared, “When developing this campaign, it was important to us that the materials feature a diverse group of individuals who are representative of the communities most impacted by HIV, including young Black and Latino gay men, as well as cis-gender and transgender women” (Fitzsimons, 2019). What Gilead misses is that although PrEP is advertised as targeting primarily queer POC, inclusion and outreach take more than online visual **representation**^5^^,^^6^. Gilead's original PrEP strategy was to portray it as an essential public health tool not a “commercial opportunity” as expressed by Gilead's spokesperson Cara Miller in 2015 (Chen, 2015). Today, Gilead is heavily invested in advertising PrEP, yet, as an example of its marketing genius that disguises commercial gains under the roof of public health, the company says, “TV advertising is a natural evolution of efforts to educate people about risk factors and what they can do to protect themselves” (Tindera, 2018).

I would like to go back to the *HealthySexuals* to raise a few urgent questions. HealthySexuals is a web platform created by Gilead, although the visitors, unless they scroll all the way down where they can spot Gilead's logo, would not notice the origin at first sight since the corporate identity behind the platform is carefully veiled to make it more user friendly. The platform invites everyone to “find [their] healthysexual side” and informs them that “there are things everyone can do to help protect their sexual health.” The homepage welcomes visitors with a brief, minute-long video, where PrEP is only mentioned toward the end of it, probably to avoid to be registered by visitors as an aggressive advertisement. The HealthySexuals supposedly gives the message of protection and its sole purpose is to provide information on sexual health, which, to the trained eye, is just another way of advertising. What the HealthySexuals campaign is not capable of asking—so I will ask for Gilead—is what does it take to be healthy? Is PrEP enough if one cannot even afford healthy food and basic medical care? The HealthySexual campaign encourages people to “be sexy and healthy” and to “talk healthy,” fetishizing health as a commodity required to be sexually attractive while, at the same time, pretending as though being healthy is simply a personal choice.

The individualization of responsibility not only for health but also for risk (Thomann, 2018; Nicholls and Rosengarten, 2019) is a conspicuous example of how pharmaceuticalization and neoliberalism are inextricably intertwined. In reference to the popular PrEP campaign implemented in the NYC in 2015 that encouraged gay men of color to “stay sure” and “play sure,” Thomann (2018) discussed the pharmaceuticalized neoliberal sexual actor who must assume exclusive responsibility for his sexual health. Consequently, responsibility, when located in the individual, is avoided by public and private institutions. What HealthySexuals campaign points out is yet another way in which the neoliberal pharmaceutical regime creates pathologies through depoliticization of health. Whereas biopolitics is about politicization of health, pathopolitics is about its depoliticization. Being able to price a medicine at about $2,000 per bottle requires an understanding of health that is not rooted in social justice or politicized. Under pathopolitics, health is treated as a product of free-market whose purchase is up to the individual's discretion. In order to cover up its complacency in the unequal distribution of health, Gilead puts the burden of being healthy on the individual or offers nominal assistance. The most popular strategy it employs to distort the reality of how it reproduces pathologies is commonly known as Corporate Social Responsibility.

### Philanthrocapitalism: Saving the World Through Corporate Social Responsibility?

Amidst all the criticisms directed toward Gilead's outrageous pricing policies, two things remained stable: the increase in Gilead's earnings (Owens, 2019) and the global recognition for its corporate social responsibility. The pharmaceutical giant is extremely proud of its success in “promoting global health” and does not shy away from branding itself as a global health super hero. Gilead dedicates a meticulously curated section to “responsibility” on its official website, placed at the very upper center, where it catches the eye before anything else. The social responsibility initiatives include *Compass Initiative*, a 10-year, $100 million partnership with community-based organizations working to combat the HIV/AIDS epidemic in the Southern United States; *HIV Age Positively Initiative*, supports programs that may help improve quality of life and health for aging PLWHIV; *US Patient Access*, helps patients to access Gilead therapies accessible for uninsured individuals and those who need financial assistance; *Developing World Access*, supports the developing world to fight against HIV/AIDS and viral hepatitis usually by funding regional organizations and cheap generics produced by Indian companies to be exclusively used in low-income countries; and, *Corporate Contributions*, an example of which is Gilead Fellowship awarded to non-profits, patients advocates, and medical researchers. Gilead is also the first pharmaceutical company to join the Medicines Patent Pool whose vision is “a world in which people in low- and middle-income countries (LMICs) have rapid access to effective and affordable medical treatments and health technologies” through voluntary licensing and patent pooling. The company whose 2019 revenue was a little more than $22 billion and whose total worth is around $70 billion prides itself endlessly on having spent $300 million only in cash donations and for being chosen the leading corporate funder 4 years in a row for helping to address HIV/AIDS epidemic by Funders Concerned About AIDS (Gilead Impact Report, 2017).

In the U.S., Gilead offers limited opportunities for uninsured people and people who are insured but have to pay co-pays. On the popular Facebook group *PrEP Facts: Rethinking HIV Prevention and Sex*, created by Damon Jacobs, a self-proclaimed *PrEP warrior* dedicated to mainstreaming PrEP, one can find numerous posts by gay men sharing their happiness with the Gilead Co-pay Assistance Program (or CAP, from which I also benefit to avoid monthly co-pays for my ARV medicine). Only those who are privately insured are eligible for CAP and can benefit from up to $7,200 annual help with drug coverage. It must be noted that this is a common practice among drug manufacturers—I am personally enrolled in two other co-pay programs offered by Jannsen and ViiV. Sadly, Medicaid participants are not eligible for Gilead assistantship. Although states that have expanded Medicaid cover Truvada for PrEP, the co-pays and other treatment-associated costs—transportation, visits etc.—can still be a huge burden for many. As Allen et al. (2017) observed, “insurance alone may not translate into access to health care” as substantial barriers exist even for the insured due to patient-level (family/work barriers), provider-level (perceived discrimination etc.), and system-level (coverage, financial, and access barriers) factors.

Ecks (2008, p. 178) convincingly exposed that strategic mechanisms such as assistance programs are inherently insufficient and employed to “distract from less obvious market mechanisms” that create the need for assistance programs in the first place. The drug donations and assistance programs have been also criticized for justifying monopoly, not being sustainable or reliable, and for pharmaceuticalizing disease and depoliticizing health (Rajan, 2017, p. 190). Žižek (2006) opines that the real evil of corporate responsibility is hidden in its ability to offer a fictitious moral action without structural transformation. Notwithstanding, as though the solution was to offer more financial assistance, on July 2018, Gilead raised the annual limit on the CAP from $4,800 to $7,200, which was widely celebrated by the PrEP-warriors.

The good news came right before Gilead announced a potential price increase of 4.9% for Truvada (Rivas, 2019), which barely found any coverage within the mainstream LGBQT media outlets. What did attract attention was the deal reached with Gilead following Donald Trump's State of the Union Address in February 2019, where he pledged to end HIV in the US (The Lancet HIV, 2019). According to the agreement, which came a few months after the US government sued Gilead, Gilead is to donate 2 million bottles of Truvada per year for up to 11 years. The donated bottles will be distributed by a new federal program called Ready, Set, PrEP—yet, the patients will still be responsible for paying for the regular blood tests and medical visits. This seemingly beneficent step taken by Gilead was rightfully called an “empty gesture” by a 2019 Lancet Editorial, which concluded, “The donations from Gilead […] are on the surface positive steps, but they will not close the gap in the number of people at risk and the number of people on PrEP sufficiently to counter the inequity in access to this proven public-health intervention.” Besides, the CDC suggests there are 1.1 million people in the US right now who could benefit from PrEP and the amount donated would cover <200.000 individuals. In a context far away from the US, Whyte and her colleagues' work on Uganda revealed that price cuts by the big multinational pharmaceutical companies, action research programs, donor support, and even the production of cheaper generics are never sufficient to provide universal access to ARV (Whyte et al., 2006). Without significant regulation of drug prices, access to medicine will never be universal neither in the U.S. nor in Africa and that it was never meant to be.

“It is sadly ironic,” Susan Craddock (2017, p. 58) wrote in her latest book, “that pharmaceutical companies might now profit socially if not financially from the disease burdens they helped create through their own strident pursuit of pharmaceuticals with hefty financial returns to the neglect of public health.” The quote from Craddock reveals what is at the heart of pathopolitics: the pharmaceutical industry contributes to the emergence of pathologies it claims to cure. If the unreasonable pricing of medicine is how the pharmaceutical industry perpetuates suffering nationally, the prevention of access to generic medicine is what globalizes suffering. The 1995 TRIPS (Trade Related Aspects of Intellectual Property Rights) Agreement signed by the World Trade Organization is the quintessential reason behind the worldwide lack of access to generic medicine. The last step of the Uruguay Round of the General Agreement on Tariffs and Trade (GATT) was TRIPS whose mastermind was the U.S. government and the pharmaceutical lobby. It was the same pharmaceutical lobby of 41 companies who pressed charges against South African Government in 1998 and criticized president Nelson Mandela for trying to universalize ARV access through generics. TRIPS is causing hundreds of thousands of people to suffer numerous illnesses and face death. Hickel (2012, p. 526) argues, “The bulk of Swaziland's present AIDS burden can be directly attributed to constraints imposed by the TRIPS agreement and the resistance of the WTO and pharmaceutical companies to changing it.”

When it comes to Gilead's CSR, there are two critical questions to be answered: Why aren't generics made available to U.S. patients and why does Gilead provide cheaper generics or donate medicine abroad? In response to the second question, Ecks (2008) claims some medicines are never meant to be affordable in the Global South. While losing the chance to exploit potential local medical markets, pharmaceutical companies win two other battles by donating medicine: they protect their good image and maintain the higher prices in the Global North (Ecks, 2008, p. 177). In essence, through drug donations and third-party generic agreements, not only will Gilead enjoy control over the locally produced drugs (Ecks, 2008) but it may partially prevent or delay any backlash from poor countries, which, as Melinda Cooper (2011) suggests, might end up igniting the public in the U.S. as well. Besides, Gilead might be rightly concerned about the lack of access to treatment in Africa. HIV/AIDS in Africa is a global concern and turning a blind eye to this would simply be a bad marketing strategy for the company who owns most of the patents on ARV medicine. One of the most important functions of CSR is to transform the conventional monstrous, greedy image of pharmaceutical companies. Or, as Fortune's *Change the World* list claims (Anderson-Minshall, 2016), Gilead can simply be a force for good (note the irony here).

As for the absence of generics for the U.S. citizens, Gilead's partners in crime are the insurance companies for profiting from the lack of universal healthcare and the U.S. government for failing to implement a functioning healthcare system. When Daniel O'Day, the CEO of Gilead Sciences, was asked during the congressional hearing to explain the lack of generic PrEP in the U.S., he gave as pretext “the government's willingness and ability to pay, market dynamics, and the structure of insurance markets specifically related to drug delivery” (HIV Prevention Pill, 2019). O'Day went on justifying the exorbitant prices on the grounds that Medicaid insurance covers Truvada for PrEP as though it does not create an immense burden on the taxpayer, who paid for the PrEP research in the first place. This pervasive normalization of lack of free healthcare and its domination by the insurance industry deceivingly moves the discussion away from those factors and agents that make it possible for the reign of the pharmaceutical companies. The insurance industry is among the principal actors who impeded the implementation of compulsory healthcare during the early 20th century (Hoffman, 2001). “American values” and capitalist market dynamics must also be accounted for here as state-sponsored healthcare was widely attacked based on its “un-American” nature that goes against the principles of free market (Hoffman, 2012). The most striking aspect of O'Day's response is how he verbalizes a dangerous open secret when he mentions “the government's willingness to pay.” Examples such as Brazil, South Africa, and Turkey make clear that the pharmaceutical regime is not stronger than people and their lives when the governments take the necessary actions to put citizens' needs before the profit of pharmaceutical companies. As corrupt as it is, the system is not broken. It “works quite well at what it is designed to do—provide a good return on investment” (Tomes, 2016, p. 416).

The question is whether “global corporate citizenship is not a brake in free-wheeling capitalism, but rather a strategy of extending and accelerating it by new means” as provocatively suggested by Ecks (2008, p. 178) or whether “the seemingly contradictory goals of ethical action and profit incentive are not mutually exclusive” as incisively pointed out by Craddock (2017, p. 57). Rajan too wrote that “ethics can be potentially opposed to surplus value but also deeply tangled within its logic” (2017, p. 21). According to him, ethics are not irrelevant but inherent to the extraction of value as it is materialized in the idea of corporate responsibility. Although Rajan is hopeful about the embrace of ethics by corporations and writes “one could envisage a value that is not just defining of capital but (in its ethical registers) also an alternative normative framework to capital,” he is well cognizant that “corporations are perfectly capable of enfolding these concerns into their own value-generating enterprises” (ibid).

## To Swallow or Not To Swallow: Pharmaceutical Citizenship and Pathopolitics

“Being poor, being black, being of color puts your life at risk. Your health is compromised when you do not have the external resources to support a life in all of its contingencies. And then of course, you are deemed responsible for your own ill health, for your own failure to look after yourself better. When you refer to structures, to systems, to power relations, to walls, you are assumed to be making others responsible for the situation you have failed to get yourself out of. ‘You should have tried harder.’ Oh, the violence and the smugness of this sentence, this sentencing.”

Ahmed (2017, p. 238)

In a piece called *chemical condoms* written in response to mainstreaming of PrEP, Preciado highlights the purpose of PrEP is not to improve consumers' life but to exploit them by creating an illusion of freedom [from fear] and liberation [from condoms] (2015). In contrast, I argue that the purpose of PrEP is precisely to improve consumers' life as long as they are able to consume and generate profit. The pharmaceutical industry cares about human life insofar as it produces a surplus value that can be extracted to accumulate wealth. In the words of Rabinow and Rose, pharmaceutical companies seek to “develop and maximize targets for pharmaceutical markets and other health-care interventions […] in the name of the maximization of quality of life” (2015, p. 317). It follows that only those bodies that can be transformed into profit-making machines deserve a quality life, as is shown below. On the other hand, having one's life quality maximized comes with its own costs. “There exist biopolitical [or, pathopolitical] side-effects (in addition to physiological ones) to mass compliance with pharmaceutical mandates” (Dean, 2015, p. 234). In return for the protection the pharmaceutical industry offers, it expects full cooperation which necessitates complicity in distributing and relocating pathologies.

**Figure 1:**
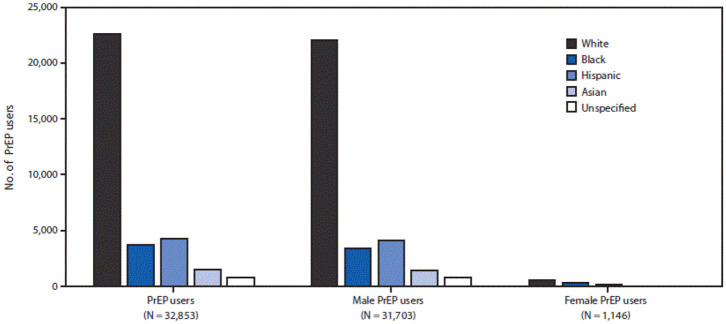
Number of PrEP users by sex, race/ethnicity-IQVIA Longitudinal Prescription Database, United States, 2016. Adopted from Huang et al. (2018).

**Figure 2:**
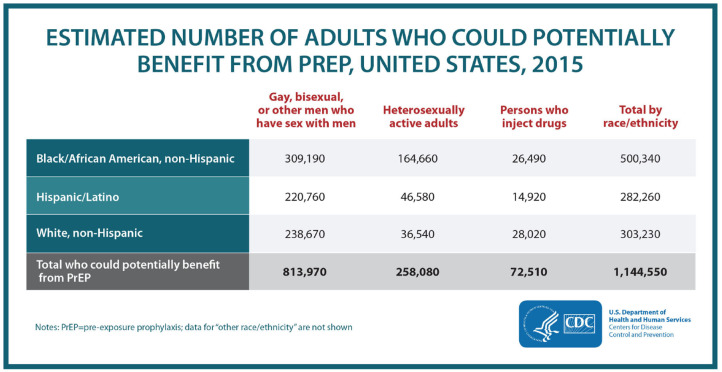
“HIV prevention pill not reaching most Americans who could benefit—especially people of color.” Retrieved from https://www.cdc.gov/nchhstp/newsroom/2018/croi-2018-PrEP-press-release.html.

Figures 1, 2 provide evidence that when it comes to PrEP what is at stake is not so much public health as it is profit (as well as pleasure). PrEP is disproportinately enjoyed by white gay men and celebrated for eliminating the need for condoms. In the words of Race (2009, p. 15), PrEP is “emblematic of a broader technology of power that converges on embodiment, consumption, and pleasure in the name of health.” The popular Facebook group mentioned earlier, *PrEP Facts*, is a perfectly suitable platform to follow the trends on PrEP use. With over twenty thousand members from all over the world but mainly the U.S., the posts on the page can be gathered under two broad categories: posts made by members who need guidance to access PrEP and stories about sexual liberation achieved a result of saying goodbye to condoms without fear (Race, 2018). One of the most common activities in the group is to create polls to see who is still using condoms and who is only practicing bareback (condomless) sex. The results always lean toward the latter. One particularly attention-grabbing post was about a gay man asking others' opinion on whether PrEP provides enough protection to fulfill his fantasies of being a “cumdump,” where multiple men ejaculate inside the same person. This post was welcomed by others who enthusiastically assured him that the beauty of PrEP comes from its ability to make one's fantasies come true^7^.

Duggan (2003, p. 50) defined homonormativity as “a politics that does not contest dominant heteronormative assumptions and institutions, but upholds and sustains them, while promising the possibility of a demobilized gay constituency and a privatized, depoliticized gay culture anchored in domesticity and consumption^8^”. The homonormative gay man is the henchman of the neoliberal state: he is an exemplary citizen because he protects social norms rather than questioning them. He is an indispensable part of the workforce, a zealous supporter of consumerism, and is patriotic. Ironically, it was the AIDS epidemic that gave way to an epidemic of assimilation. In conjunction with public health discourses and prevention technologies, gay men are made into “proper” healthy citizens, who are monogamous, ideally married, or practice only safe-sex and remain HIV- at any cost (Davis, 2002; Keogh, 2008; Gonzalez, 2010; Robinson, 2014). Thanks to their surplus economic and biopolitical value, they have taken their place among those whose lives matter and shall be protected, even at the cost of others. As Collins (2009, p. 467) wrote “homonormativity—like heteronormativity—is an exclusionary process; inclusion is for select bodies—white, middle-class, consumerist, Western, and often gay male bodies who have access to the consumer “freedoms” of the West.” In Stefan Ecks' words, the homonormative gay man is the most desirable citizen under the framework of “pharmaceutical citizenship” which not only determines who has the right to access medicine but also operates in a feedback loop such that those who take the medicine become more fully citizens (Ecks, 2005, p. 241). Thanks to PrEP, gay men can now enjoy condomless sex without risking HIV or losing their citizenship privileges.

Even though the original conceptualization of homonormativity puts a lot of emphasis on the intimate relations between queer subjects and state institutions such as military and marriage, what I want to call attention to is another set of relations and practices quintessential to the operations of the pharmaceutical regime. Queer citizens today extend the realm of homonormativity to the uncritical consumption of pharmacological discourses and products, therefore, contribute to pathopolitics. Gay men's contribution to the extraction of surplus value is not limited to their consumption and labor. Neither can it be reduced to their enthusiastic advertisement of PrEP, which is claimed to be the most effective form of pharmaceutical advertising (Elliot, 2010). They also produce infinite value through what Preciado (2013, p. 36) calls *masturbatory cooperation*: every excitation and every ejaculation achieved on PrEP extends the reach of biopower and the revenue of pharmaceutical industry. White gay men's HIV negative cum is not wasted knotted up in latex condoms in the garbage, but, rather turned into a profitable asset through PrEP, circulating not only between bodies but also in the pharmaceutical market. Already engaged in an intimate relationship with the state, the homonormative citizen opens the doors of his bedroom to the pharmaceutical regime and invites it to be a part of and enjoy the most intimate bodily moments. And, he does so willingly without being coerced by the state. “It is not power infiltrating from the outside,” said Preciado, “it is the body desiring power, seeking to swallow it, eat it, administer it, wolf it down, more, always more, through every hole, by every possible route of application” (2013, p. 208). In this consensual encounter between the body and power, both of them find pleasure in penetrating and being penetrated.

Pathopolitics does not only determine who gets to live disease free but also who gets to enjoy sex without risking HIV infection. When it comes to women, trans persons, people of color, sex workers, substance users, and HIV+ people who are not on medication, their orgasms are not equally valuable or lucrative. Preciado writes: “the new hegemonic subject is a body (often codified as male, white, and heterosexual) supplemented pharmacopornographically (by Viagra, coke, pornography) […]” (2013, p. 48). To this description, I would add that a new hegemonic subject is the white gay man who is supplemented by PrEP. The security and protection provided by PrEP is nothing new for the homonormative subject who benefits from all the material and immaterial advantages of being privileged. “When a whole world is organized to promote your survival, from health to education, to the walls designed to keep your residence safe, to the paths that ease your travel, you do not have to become so inventive to survive,” wrote Sara Ahmed (2017, p. 237) powerfully in another context. You do not need to be inventive to survive; if not the state, then the pharmaceutical companies will find a way to keep you alive, so long as you keep producing profit. This is by no means to deny the problems even the homonormative subject can face. “Privilege does not mean we are invulnerable: things happen; shit happens. Privilege can however reduce the costs of vulnerability; you are more likely to be looked after” (Ahmed, 2017, pp. 237–238). Even though not heterosexual, he is still cared for and made to live by the same system that condemns marginalized people to slow and not-so-slow violence and death. PrEP is only another piece of the larger puzzle, extending economic, political, and social safety into corporeal satisfaction and biological security. It is through such improvements the bare flesh becomes a fully abled social subject, blurring the lines between bios (qualified, meaningful life) and zoe (unqualified, bare life) (Agamben, 1998). It is not the life alone that matters anymore; it is a particular way of life—a more sexual, more aroused, more commodifiable and marketable one, where bodies are more fuckable. It is less about bare life than it is about bareback sex.

Lastly, the final question is what kind of sufferings and pathologies are produced in the making of some bodies more biosecure and sexually attractive? To put it another way, whose suffering made the consumption of PrEP possible? The pharmaceutical industry complex does not simply cure pathologies; instead, it relocates them. The prevention of HIV for the citizens of the Global North might mean exposing the disposable bodies of the Global South to increased risk of HIV. One could say some are sacrificed so that others can enjoy more pleasurable and less risky sex. The pharmaceutical industry produces global casualties by recruiting “treatment-naïve” populations found in resource-poor countries, where trial recruitment and conduct is less costly and less time-consuming due to insufficient regulations and monitoring (Petryna, 2006). The first PrEP trials in Cambodia, funded by NIH and Gates Foundation and not by Gilead^9^, were conducted with sex workers. Nevertheless, they were halted in 2004 by the Cambodian Prime Minister. Among the reasons that incited widespread demonstrations by small local HIV and queer activist groups were inadequate prevention counseling, a lack of pre- and post-HIV test counseling, non-provision of services for those who seroconverted during the trials, insufficient data about the long-term effects of tenofovir for HIV- people, and the inadequate involvement of target populations in the research design and implementation. As the activist groups made clear, “participants take all of the risks and get little [if any] of the benefits” (Singh and Mills, 2005). In 2005, trials in Cameroon were canceled due to similar concerns about lack of counseling. Yet, local activists this time made an astonishing claim about participants being intentionally exposed to risk of infection (ibid). Unlikely though it sounds, the case of Cameroon uncovers a constitutive failure of global health and randomized drug trials. Researchers most often find themselves trapped between meeting ethical standards and obtaining “desired” scientific outcomes (Adams, 2010). Obtaining the most profitable outcomes, although not necessarily the most scientific ones, might at times require manipulation of the data (Dumit, 2012). It may too require giving placebos—as is the case in PrEP trials—to members of poor marginalized populations and watch them become infected with HIV. Years after the first PrEP trials, the trend of outsourcing human subjects has remained the same. Among the countries where the succeeding trials were conducted are Kenya, Uganda, Thailand, Botswana, Peru, Ecuador, and Zimbabwe, most of which suffer from the absence of universal access to ARV treatment. It must be noted that the ethical issues with PrEP trials are hardly only about the outsourcing of research participants. In an article entitled The Cost of Science, Patton and Kim (2012) question the ethics of PrEP trials altogether. They argue that PrEP trails used the limited resources for pharmaceutical interventions instead of community support and divested resources from people who already live with HIV. Patton and Kim also strongly defend that neither were trial results transferable to the U.S. nor they were able to prove enough efficacy for the use of women (which was ignored for the benefit of MSM). Their most controversial point is on the potential misinterpretation of data, which might have obscured how PrEP can do more harm than good.

## Conclusion

As I was finishing this essay, the COVID-19 pandemic hit the world, which like any other modern epidemic or pandemic meant disaster for those affected and business for those who profit from disasters. Disaster capitalism can be observed at its worst when human life is at stake. Gilead was among the first scavengers who rolled up their sleeves to benefit from the pandemic. One of Gilead's broad-spectrum antiretroviral medicine, Remdesivir, also developed with US government funding, promised hope against the novel Coronavirus (Fang and Lerner, 2020). As a result of high demand, on March 23rd, Gilead announced it would stop providing emergency access to Remdesivir. Following the announcement, the drug was given orphan status by the FDA within the same day (ibid.). Orphan status, which gives the manufacturer the exclusive control of the drug and its pricing, is reserved for drugs used to treat rare diseases that affect fewer than 200.000 individuals. However, due to a loophole, popular drugs can enjoy orphan status if they earn it before the disease reaches the threshold. This was the case with Remdesivir and it visibly increased Gilead's stock price in a matter of hours (ibid). The story of Remdesivir is but an example of how much can a drug company value profit over life during extraordinary circumstances. The only thing that separates this story from others is that on March 25th, following widespread public outcry, Gilead surprisingly announced it will seek to rescind orphan drug designation for Remdesivir (Lerner, 2020). There is a limit, an invisible line, the pharmaceutical industry sets for itself to judge how much of greed is too much. It turns out it is not yet too much to exclude 48% of global population—including low- and middle-income countries—from the geographical scope of the voluntary licenses Gilead provides for the production of Remdesivir's affordable generics (Baker, 2020). Neither has the limit been breached yet when Gilead was blocking generics and setting unaffordable prices for a life-saving Hepatitis C drug, Sovaldi, only a few years ago (TAG, 2015).

Gilead is but one example of countless other pharmaceutical companies that pit the right to health and life against the right to make profit. The monopoly over patents bestows drug companies with no public accountability the monopoly over the distribution of risk. Instead of relinquishing the monopoly, the companies would rather donate drugs or provide aid, which they then call corporate social responsibility. Writing about Novartis' resistance to renounce its monopoly over the anti-cancer medicine Gleevec in India, Rajan expressed, “The limited responsibility of corporatized philanthropy sits comfortably with an idea of Responsibility Ltd. It is a form responsibility that is completely appropriable and appropriated by the interests and instruments of global capital” (2017, p. 238).

Pathopolitics, as I argued in this essay, is the corporate politics of strategic distribution of pathologies and suffering. Drug companies develop and manufacture technologies to be used to remedy or prevent pathologies. Nevertheless, they strengthen the existing pathologies, or create new ones, by making these technologies accessible only to a few. Through unjust pricing policies and aggressive control of generics, the companies aggravate pathologies and the suffering they cause. In addition, the suffering of some has increasingly become the necessary condition for the treatment of others. The pharmaceutical industry is producing pathologies for certain populations precisely to cure or protect others, who promise financial returns. PrEP unveils the way in which the distribution of pathologies is determined by how much surplus value individuals can offer. One of the questions this article sought to raise is whether those of us who use drugs to prevent pathologies are to a certain extent complicit in pathopolitics, which does not so much do away with pathologies as it relocates them to other parts of the world, away from where they can be seen or heard. What PrEP lays bare is that health and sexual pleasure might come at a cost: the uncritical advocacy and consumption of a medicine that is by nature exclusionary and discriminatory might inadvertently reinforce pathologies, social and biological.

Given that pharmakon means both poison and cure, the central paradox of pathopolitics lies in how the pharmaceutical industry sometimes poisons so as to cure: it promises to treat not only the existing pathologies but the ones it helped create. The way it does that is called Corporate Social Responsibility, which aims to balance two sides of pathopolitics. As the case of Gilead reveals CSR functions akin to putting a cheap band-aid on an infectious wound in need of medical attention—under the bandage, the infection will keep spreading to the point where it could become lethal or cause the mutilation of a limb. Pathopolitics, hence, is not kept in balance by CSR but, rather, turned more destructive, more pathological.

## Data Availability Statement

The datasets generated for this study are available on request to the corresponding author.

## Author Contributions

The author confirms being the sole contributor of this work and has approved it for publication.

## Conflict of Interest

The author declares that the research was conducted in the absence of any commercial or financial relationships that could be construed as a potential conflict of interest.

## Footnotes

^1^I would like to thank Susan Craddock for offering the term “pathopolitics” to conceptualize my work on PrEP and Gilead; David Valentine, Dominique Tobbell, and Aren Aizura for their most helpful feedback; and, Nicholas Greatens for his extraordinary editorial skills. I also have to thank Karen-Sue Taussig for drawing my attention to the previous invocations of the term pathopolitics. The first instance is Mariella Pandolfi's use of the term in 2008 (see Allen, 2012) while analyzing the intertwined nature of humanitarian aid and military intervention in the case of NATO's bombardment of Serbian forces during the Kosovo War. The second is a recent dissertation (see Huber, 2017) exploring “the increasingly politicized nature of the cultural representation of emotions in contemporary American culture.” Although in both instances Foucauldian biopolitics is what inspired the authors' use of pathopolitics, I am employing the term with completely distinct purposes that are not inspired by either of its previous uses.

^2^It is reported that Larry Kramer later changed his opinion on PrEP and recognized its potentials, while still being unapologetically critical of Gilead.

^3^According to an investigative report published in 2016, Gilead avoided paying almost $10 billion in taxes thanks to untaxed offshore profits (Merle and Johnson, 2016).

^4^Gilead's new TV ad for Descovy is called “Prep Up, Step Up.” The meticulous performance of inclusivity that started with the first TV ad on Truvada, entitled “I'm on the Pill,” continued with the new marketing campaign showcasing a trans woman of color, gay men of color, and a drug queen.

^5^I personally participated in marketing research for Gilead's existing and future webpages twice in 2018. Both times, I was shown real and animated images of POC and asked my opinion on the accessibility and attractiveness of the visuals as well as the information provided.

^6^The company proudly underlines that it has invested $100 million in community-based organizations to support HIV prevention awareness (Tindera, 2018). Based on my research in Turkey, I can say that if the nature of its investment is in any way similar to its philanthropic operations in Turkey, those millions of dollars are spent less on actually strengthening communities than on the production of what can be a called a community-to-pharmacy pipeline. In Turkey, Gilead's support of local HIV organizations more often than not aims at reaching out to HIV+ persons who are not yet diagnosed to put them on treatment.

^7^I would like to open a parenthesis here to mention my purposes to evoke the figure of cumdump, which terrorized both the heterosexual and gay communities during and after AIDS crisis. The exchange of bodily fluids, especially that of sperm, is still pathologized in the context of public health, denying the crucial importance of carnal pleasure to people's lives. Although there is virtually no aspect of gay men's sex lives that is not yet thought to be destructive both for the self and for the community, the abandonment of condoms has especially been panic inducing for almost four decades now. I use cumdump as a provocative example precisely to point out PrEP's ability to allow the materialization of fantasies and uninhibited practice of pleasures. I am in no way against the intensification or proliferation of sexual pleasures through PrEP. When I was first diagnosed with HIV, I found infinite emotional and sexual comfort in knowing that my partner could use PrEP. I still find a lot of comfort when I have sexual interactions with strangers who are on PrEP. But, this is not about me and how I find pleasure in the presence of PrEP. This is about the political potentials of pleasure. Bodies and pleasures, said Foucault (2003), are the only sites wherein which resistance to biopower is possible. If, in this essay, pleasure appears to be overdetermined by biopower, it is because I do not believe that pleasure stands outside the realm of biopower: that is, pleasure can resist biopower precisely because it operates within not outside. While critiquing the pathopolitics of PrEP, I would also like to consider the ironic ways in which PrEP can provide radical queer alternatives to normative sexual practices. Besides, I would be contributing to the biopoliticization of PrEP should I argue that it must only be used in the name of health by those who need it, distributing pleasure on a racial basis. I would be happy to see PrEP becoming a real “party drug” one day—that is, accessible to all and not only a privileged few. The unintended moral tone of my argument, therefore, does not stem from a critical stance toward pharmaceutically intensified pleasure, which can effectively disturb the cultural associations between condomless sex and self-destruction (Race, 2018). Rather, it stems from the nature of pleasure obtained through PrEP that is at once exclusionary and discriminatory. That is, pleasure, as politically subversive as it might be, comes at a cost, which is the reproduction of pathopolitics.

^8^Even while discussing how homonormative citizens expand the reach of pathopolitics, it must not be forgotten that the potential to subvert homonormativity still lies in the figure of cumdump, who resists to be domesticated and disciplined, even when on PrEP (or, maybe, because on PrEP).

^9^Note that Gilead's presence is always noteworthy within drug trials even though they are not funded by it. The company still provides the drugs for the trials, whose research teams include Gilead's own researchers.
